#  Usalpharma: A Cloud-Based Architecture to Support Quality Assurance Training Processes in Health Area Using Virtual Worlds

**DOI:** 10.1155/2014/659364

**Published:** 2014-03-20

**Authors:** Francisco J. García-Peñalvo, Juan Cruz-Benito, Cristina Maderuelo, Jonás Samuel Pérez-Blanco, Ana Martín-Suárez

**Affiliations:** ^1^GRIAL Research Group, Department of Computer Science and Automatics, University of Salamanca, Paseo de Canalejas 169, 37008 Salamanca, Spain; ^2^Department of Pharmacy and Pharmaceutical Technology, University of Salamanca, Faculty of Pharmacy, Campus M. Unamuno, 37007 Salamanca, Spain; ^3^IBSAL Salamanca Institute for Biomedical Research, University Hospital of Salamanca, Paseo San Vicente 58-182, 37007 Salamanca, Spain

## Abstract

This paper discusses how cloud-based architectures can extend and enhance the functionality of the training environments based on virtual worlds and how, from this cloud perspective, we can provide support to analysis of training processes in the area of health, specifically in the field of training processes in quality assurance for pharmaceutical laboratories, presenting a tool for data retrieval and analysis that allows facing the knowledge discovery in the happenings inside the virtual worlds.

## 1. Introduction

Virtual worlds and serious games are now a resource increasingly accepted to train skills and acquire knowledge in various areas, involving both formal learning, especially in universities taking into account new technological-based pedagogical approaches [1], and informal learning, more oriented towards workplace training and personal skills development [2]. But how are they able to successfully face the interaction of thousands of users expecting to complete these tasks? This is because of their infrastructure and architecture. Virtual worlds and 3D serious games are currently impossible to maintain by the use of classical server structures, as the user interaction within them is changing and does not respond to traditional usage patterns and resource requests. Virtual worlds and online 3D games had a radical change from the acceptance and use of the concepts related to cloud computing and cloud-based architectures, where resources are allocated and released dynamically, so they give a changing service, agreeing with the type of interaction and load they have. For example, Second Life (SL) typically has design of one or more (physical or virtual) servers to support one region or island, but this allocation varies with the interaction of the various elements within the virtual ground, so that if the region has a use peak other servers with free resources can support the tasks and improve overall response against these workloads [3–8]. Definitely, virtual worlds and 3D serious games currently base their success on flexible architectures that support many tasks in a transparent and dynamic way. Building on this, this paper tries to extend these concepts to a different use in the virtual worlds, such as support of the interaction analysis, in this case focusing on supporting cases of students training processes in the field of the health sciences.

The current generation of 3D virtual worlds began to develop about ten years ago. SL was the first to achieve popularity and a high degree of development [9]. Since 2008 many other virtual worlds have emerged with which SL has been sharing its leadership. But currently, SL is still the most widely used virtual world for healthcare and higher education activities [10, 11]. All the content in SL has been generated by the users. They can even create their own objects and spaces using software provided by SL [11].

Several authors have explored SL [6, 12, 13] and found a wide range of healthcare-related activities. SL has been used for health education, community outreach, training healthcare providers, and market and promotes health services and simulations purposes. Reference sites in healthcare are Healthinfo Island, Imperial College London, Virtual Hallucinations, or Second Health London [14–17].

Virtual worlds such as SL provide unique opportunities to simulate real life scenarios and immerse the user in an environment that can be tailored to meet specific educational requirements. In these immersive learning environments, learners and teachers can interact from anywhere in the real world [18]. SL has been used to train healthcare professionals in virtual problem solving and communication, in addition to other conventional preclinical teaching methods, prior to student treating patients in the clinical setting [19, 20]. Several experiences have been developed in SL where students could safely practice communication and assessment skills with simulated patients [21], developing clinical diagnosis and decision-making skills [22, 23], training procedures [24, 25], assessment of competencies [10, 26, 27], and interacting with 3D physiological models [28, 29]. Also SL has been successfully used as a resource for health education [30] and for continuing education for practitioners [11, 31–33] and even has been used in real therapy sessions [34, 35].

On Usalpharma island from SL, property of the Department of Pharmacy and Pharmaceutical Technology from the University of Salamanca, we have been carrying out activities to develop professional skills in undergraduate and postgraduate students of pharmacy since 2010 [36]. These activities take place in various facilities such as a community pharmacy or a laboratory for drugs quality control. Pharmacists must be the health professionals responsible for providing patient care that ensures optimal medication therapy outcomes [37]. For this purpose, the future pharmacist should be formed in many different areas such as drug design, drug manufacturing, dispensing, or treatment monitoring. In the community pharmacy of our island, students can perform role playing activities to train metodologies of pharmaceutical care that involves the identification, resolution, and prevention of potential drug related problems [38, 39]. There are also spaces on the island for meetings and presentation of papers [40].

The other key facility is Usalpharma Lab. This laboratory simulates the installations, equipment, documentation, and tools like a real quality control laboratory of the pharmaceutical industry that fulfill regulations to such effects [41]. This kind of installations in the real world is very costly and unusual in universities. Usalpharma Lab has been used for training of pharmacy postgraduates in quality assurance. Highly positive results were obtained as regards both the achievement of the educational goals and student satisfaction [42]. To carry out these practices, both avatars of teachers and students meet in the virtual laboratory. The teacher guides and evaluates the student during the activity. But it would be ideal that the student could access the laboratory whenever he wanted using it as a tool for self-training. Data generated through the student activity could be used by the teacher to control and evaluate their activities, without having to be present at the same time [43]. These data could provide information of great relevance for the design of new practical activities as well as the evaluation and monitoring of the correct implementation of them.

The aim of this study is to deploy a cloud architecture that supports the needs described in a virtual world, including the mechanisms of data recovery and analysis of data for proper evaluation of the practices developed inside. This will be catalyzed in this paper by the development of a case study with a tool destined to get data and analyze the insights retrieved from the virtual world.

The paper is organized as follows: one section for proposal of cloud architecture, another section for components and workflows of the cloud system, one section about a study case where we use the cloud proposal, and finally the conclusions.

## 2. Cloud Architecture

As discussed in the Introduction, this paper aims to articulate a cloud architecture taking advantage of data and information generated within a virtual world like SL, so starting from what happens within the virtual world we can extract and analyze information, getting to discover knowledge that serves to gain insight of what happens or provide support in decision-making processes. This way we may tackle the isolation and lack of interoperability issues that virtual worlds in general and SL in particular present; these virtual worlds usually have a structure of cloud servers, and they generally do not allow integrating them into third party software components as shown in this paper.

For example, SL or other private virtual worlds do not provide APIs or explicit methods for interaction and exchange of information with other software platforms; they only provide in-world APIs to connect with web services or retrieve information from external websites.

For this reason, the software architecture we describe should attach or wrap the private cloud architecture from virtual world, getting by this way a unique and unambiguous process for data generation, storage and analysis.

In order to define a cloud architecture to solve problems such as those discussed above, it is necessary to consider a possible definition of the context of cloud computing.

*Cloud Computing refers to both the applications delivered as services over the Internet and the hardware and systems software in the datacenters that provide those services* [44].

Taking this contextualization of the problem, when a cloud architecture is defined, it is essential to design the system so that it is flexible enough to adapt itself to any data center or server farm, regardless of location, organization, technology, or number of machines [45]. In this paper, the hardware part is not discussed, due to the fact that our cloud architecture proposal is intended to have the possibility of being deployed on any current cloud product provided by enterprises like Amazon, Google, Heroku [46–48], and so forth.

Based on the experience gained during years in the field of computing and software engineering, it is possible to base the solution design patterns and software quality factors. Our proposed architecture is based on a layer structure. The layered architecture makes each layer given different responsibilities available within the group [49], thereby forming a modular cloud system.

From these concepts we propose a modular cloud that is composed by software services (in line with SaaS concepts) [50]. Particularly, we extend the concept of software as a service (Saas), to a wider platform, obtaining concepts as CaaS (*cloud as a service*) or PaaS (*cloud platform as a service*), so each module or part of the cloud can be a cloud service or cloud architecture itself and contribute together with the other parts to achieve the common goal. As abstraction of this set of clouds within a single cloud, in the description of cloud modules, we talk about service layers or* layers*: inside these layers, we could find clouds as a service or software modules as a service.

The integration of this cloud of clouds with the Virtual World is designed thereby due to the location of SL in their own private and closed cloud of servers and resources [51, 52]. It is necessary to propose an architecture capable of interacting with it minimally, using this locked environment to generate the best final outcome for analyst, so, through minimum data output from SL private cloud, our* Usalpharma cloud* is able to maximize the final outcome and analysis capabilities. The process to maximize the outcomes with a minimal income is supported by the cloud layers, and they are responsible for the support of the whole quality assurance training process in health area. With this solution, we also try to manage the service level management in order to address dependability [53] in the privative and close approach that SL presents.

**Figure 1:**
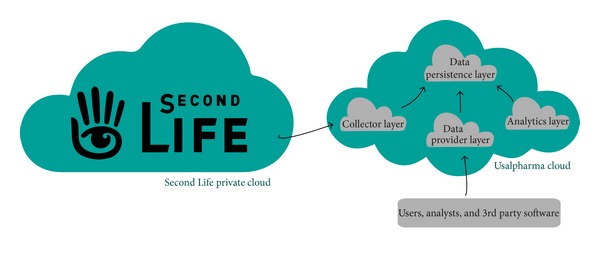
Usalpharma Cloud Architecture.

The layers for cloud architecture (named “*Usalpharma Cloud Architecture,*” Figure 1) are the following.

- *Data Collection Layer*. This layer of the cloud architecture is intended for retrieval and processing (prior to the storage) of the raw data which arrives from the users platform; in this case it arrives from the SL virtual world which actually is another private cloud of servers and software components.
- *Data Persistence Layer*. This layer organizes and stores information persistently. It is the key layer of the architecture, on which depends the proper functioning of the cloud system. It needs to be flexible enough to accept the storage of heterogeneous data coming from the virtual world. This layer must also provide a sufficiently powerful (without bottlenecks) system so that any layer of the cloud architecture can refer simultaneously with others without affecting performance.
- *Analysis Layer*. This layer is responsible for performing tasks more complex or costly in time analysis. It communicates with the persistent data layer and launches processes and algorithms to discover relationships in the data and analyze the behavior and interaction of users of virtual world.
- *Data Provider Layer*. This layer is responsible for processing data requests coming from users and analysts or other 3rd party services that can be connected to our cloud. It manages interaction with the persistence layer by any data request.

## 3. Workflows and Components

In order to achieve a correct collaboration between software layers, or cloud modules, we need to establish which components may be the components of cloud architecture and which workflows can exist between them.

Below the components and workflows of the cloud architecture are detailed briefly, emphasizing the specific part of how the cloud environment could be built.

*Components*

- Web layer to collect data: the data collector layer is intended to be built based on web services, so through API requests (URI request, etc.) the data could be aggregated to cloud environment.
- Database servers or cloud database to give a persistence layer and ensure access to every component in cloud architecture: this component is the central part of the cloud architecture and is used by all other components. It must guarantee the correct interaction and usage load by other components and ensure the integrity and proper preservation of the data. At present there are many database servers and storage software, each having different features, one or the other of which can be recommended depending on the technical requirements of the system. In the case of a cloud as the one proposed architecture, there are three key features that must be met; the system must ensure data persistence storage of heterogeneous data, the correct system scalability, and load balancing. Support for heterogeneous data is because the information coming from the virtual world can be of different types and vary over time depending on the events and interactions that are collected, so that the right support heterogeneous data (not only in storage but features such as indexing, search, retrieval, etc.) will ensure the full support to the training processes in the health area. The features of scalability and load balancing in data persistence layer ensure that the data growth and aggregation over time are performed correctly, thus allowing the data persistence layer is capable of supporting each once more data load and adapt to the demands of use in both the present and the future [54].
- Web layer to provide information: this component should provide data on demand to the analyst or third party service. It must be able to properly access the system data persistence and above all to provide the data analyst with the way required by this want. Therefore, this component must have a specific API or software methods by which the analyst or software can define the data and the presentation that he or she needs.
- Software services or cloud services to analyze data storage at database: this component is one of the most complex cloud architectures. In it lie the logic of analysis and therefore much of the usefulness of cloud architecture and the logic support of the training process that takes place in the virtual world. Within this component many algorithms and logical processes of data analysis can be found. Depending on the objectives the analysis must support different collections of methods so that they can be applied separately or together. Due to the complexity of these tasks (cost in time, CPU cycles, etc.), the executions can not be raised on demand and must be performed automatically, saving the results in the data persistence layer, so they can be picked up later by analysts or when software processes require them.

**Figure 2:**
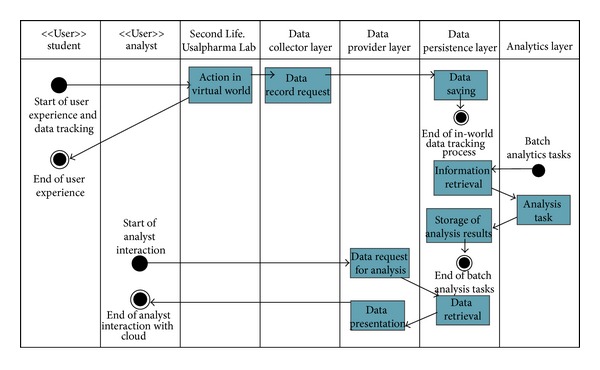
Activity diagram for Usalpharma cloud.

*Workflows (represented in the activity diagram of Figure 2)*

- Workflow for data collection is as follows.
  - User interacts with the virtual world, using the 3D scene and the theoretical concepts behind this training method.
  - From virtual world (SL) to data collector layer: we use a data model for interconnection between virtual world and data collector layer based on RDF specification [55]. The data model has a structure* subject + verb + predicate *(*user + action + object and interaction data* such as time). This data model allows the achievement of some goals, such as having a simple data model or having formal semantics and provable inference (e.g., for implementation of semantic analytics in the cloud) or even the possibility of using an extensible URI-based vocabulary (in the same way of part of RDF goals described in W3C specification). Requests are made via HttpRequest, containing in the URI the data as explained previously.
  - Data collector to persistence layer: this communication is made with the concrete API of the data persistence layer technology. The collector component composes the data with the exact method and sends it to database layer.
- Workflow for scheduled analytics tasks is as follows.
  - Analytics layer executes batch actions in order to get new knowledge from data storage in databases. For this purpose, it retrieves information from database, using the software methods according to storage technology. Later it executes the analytics tasks, by applying algorithms of data mining, statistical methods, Map/Reduce strategies [56, 57], and so forth.
  - Finally, the results of the analysis tasks are stored in the persistence layer, so that they are recoverable by analysts.
- Workflow for analysts is as follows.
  - User/software service requires data for analysis sending HttpRequests to data provider layer. This request may contain heterogeneous information about users involved in the interaction, objects, actions, date and time, and so forth. The request may also contain information about the format of the data returned from the request. This allows them to filter and get results in a more accurate way.
  - Data provider layer uses the data petition to compose a new petition for data persistence layer, which will return the data requested.
  - Finally, the data retrieved are sent to user or software in the way they require in the HttpRequest made at the beginning of the workflow.

## 4. Case Study

The case study presented here is based on the application of the cloud architecture described above to the case of a training process in quality assurance carried out by using a 3D environment as a virtual world like SL. Using data generated by user interaction, we propose the application of the system for collecting and analyzing data to help understand the process of training of pharmacy postgraduate students in quality assurance questions.

Our training scenario in this health area, Usalpharma Lab, is a pharmaceutical research and development (R&D) laboratory constructed in accordance with the quality standards demanded in the pharmaceutical industry. This kind of laboratories must meet current Good Laboratory Practice (GLP) norms. GLP refers to the set of regulations, operative procedures, and practices established by certain agencies, which are considered to be essential to ensure the quality and integrity of the data generated in different types of research or lines of study [58].

**Figure 3:**
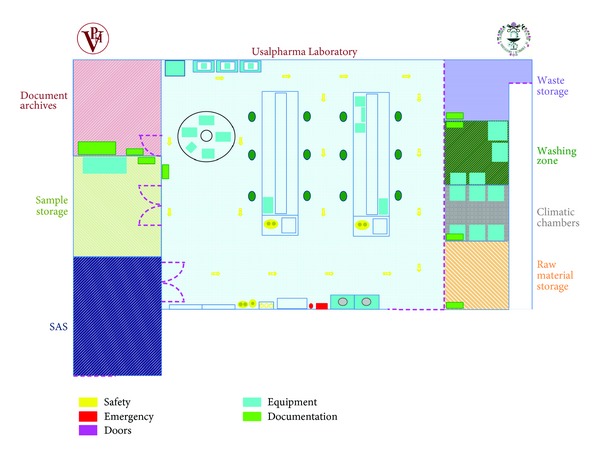
Usalpharma Lab layout.

The layout of the laboratory is shown in Figure 3. According to this layout, the access to the laboratory is a special airlock system (SAS) that leads to the main laboratory area. Around the main area are different rooms such as document archives, storage, and production of purified and ultrapurified water. In sum it has much of the equipment and materials found in a real laboratory of this kind.

This laboratory has been built for training postgraduate students in quality assurance. Different scenarios are simulated in the installation in order to test the students auditing techniques knowledge. The students should follow a checklist with different items to audit, and they should identify regulation deviations alluding to the specific regulations aspect and should establish a classification of such deficiencies, depending on their criticality. Until now, all of the regulation deviations must be reflected in an audit report with a final decision about the adaptation level of the laboratory to the GLP regulation. Our goal is to use the cloud platform to improve the insight of the auditing process made by students, in order to allow these students to perform the activity without the teacher and anytime. For this reason, we propose a set of control points that will help us meet the audit system followed by them (Table 1).

Applying the cloud architecture, the teachers evaluation system could comprise both the audit report made by the student and all the interactive activity carried out for him into the platform. The report should reflect the student's assessment of the regulation compliance and the information arising by the cloud system allowing the teacher to establish the traceability of the student activity into the laboratory. So, combining the report and the cloud data, the teacher can control and evaluate the activity in a total way (Table 2).

In order to perform a proof of concept of the proposed cloud architecture, we have implemented part of it, providing a minimal system that collects data from the virtual world, performs basic analysis that allows achieving the goals proposed to obtain knowledge about interaction, and provides a system for search and retrieval of data from analysts and teachers.

To develop the proof of concept, we have made use of three main technologies: LSL (Linden Scripting Language) [59], Django Web Framework [60], and MongoDB [61]. We use LSL to send data from virtual world (from the SL private cloud); this communication is made in only one way and is made by only one request; this is so, to avoid the latency in virtual world. Latency is the enemy of games or virtual worlds, and the information transmitted from the virtual world to cloud architecture must fit into a single packet wherever possible [4].

We use Django Web Framework to develop several web applications and services, which allows articulating the different components of the cloud system. Finally, we use MongoDB database to perform the data persistence layer, because this database meets the premises of support heterogeneous data, correct scalability, and load balancing [61] that we discussed in the section of* Workflows and Components*.

Once we implemented (and deployed) the proof of concept with these technologies, we performed a series of tests and pilots with users, so that they could test the proper operation of this minimal system built.

**Figure 4:**
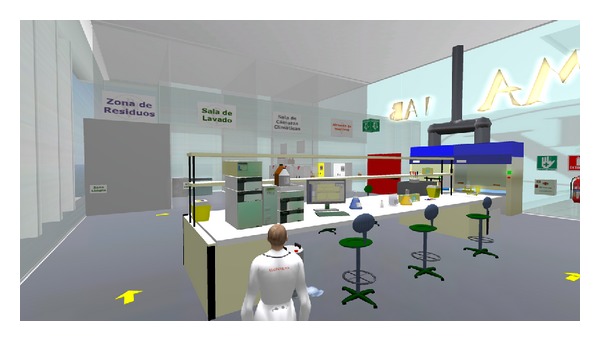
Snapshot of a tester during the pilots.

The tests were carried out for 5 postgraduate students in pharmacy, so that they perform the audit training process (Figure 4), but without having to deliver the final report on the defects or regulation deviations.

**Figure 5:**
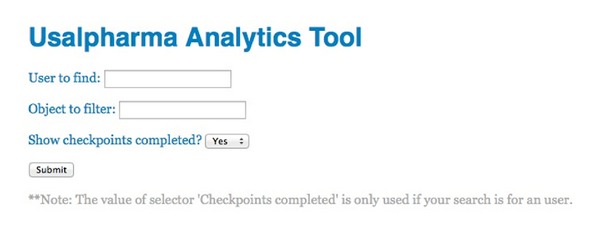
Web application to request data for analysis.

To verify proper collection and use of analysis tasks we implemented a small web tool (Figure 5) to search and retrieve results data stored in the cloud architecture (through communication with the provider data layer).

**Figure 6:**
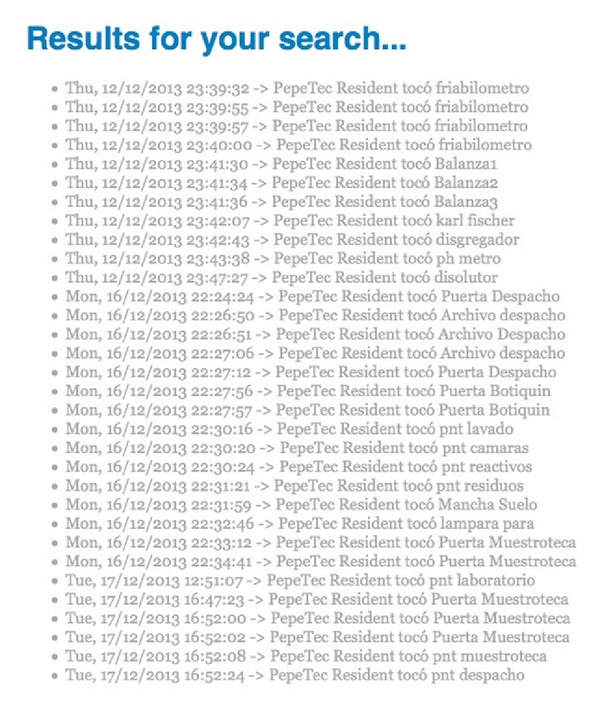
Results for user search.

As seen in Figure 5, we could perform various types of search, filtering by user or by object, and we can select if we want to read the checkpoints achieved by the user. For example, if you run a search for a user to check their interaction with the 3D environment (without considering the evaluation of checkpoints), we can get a result as shown in Figure 6. The results include the user, the interaction performed, the object that receives the interaction, and the date and time when the action was performed.

**Figure 7:**
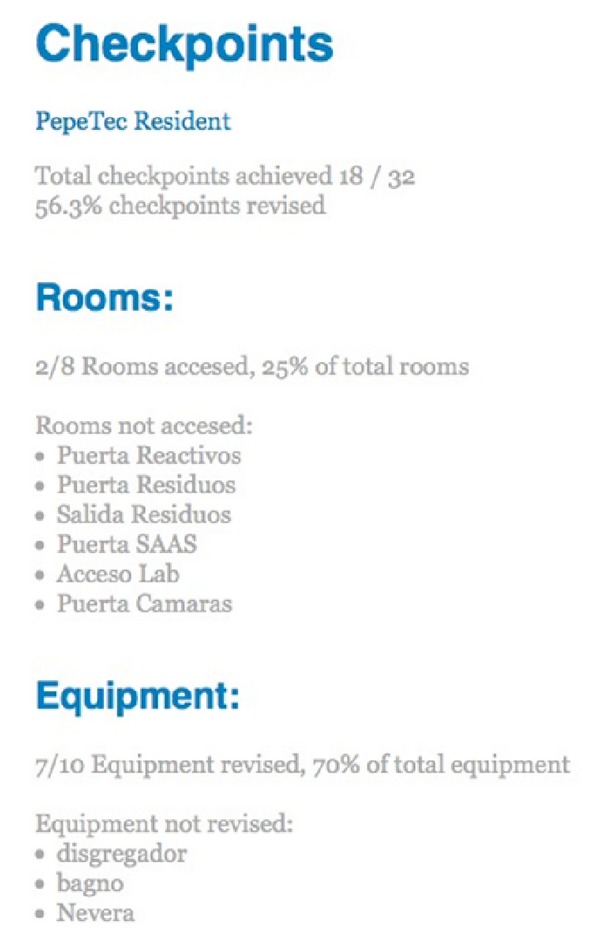
Search for checkpoints achieved by one user.

If we select the checkpoints information in search results, we can get results quite similar to Figure 7. These results include the number and percentage of checkpoints achieved by the user and which of them (clustered by types of items to audit) the user did not complete.

In light of the results, we can say that, through this cloud architecture for recovery and analysis of data, we can get a lot of detailed information about student activity within the virtual world, which otherwise is almost impossible to obtain in virtual worlds like SL. This solves a problem observed in this type of practice in a Virtual World [43]: the teacher should be virtually present during the entire activity. Moreover, it is very difficult to review what are doing all students in the laboratory at same time. Therefore, applying this or other system to support the detailed control of user interaction, can be capital to organize activities such as this, even increasingly complex training and allowing to obtain better results.

## 5. Conclusions

Throughout this paper we have specified a complete cloud architecture, starting from abstract design to workflows and components and getting to perform a proof of concept applied to a real case training audit quality assurance in the field of health care.

We consider that this cloud architecture allows carrying out the activity without the teacher's presence during the students practice. This is important to open new possibilities in training field. In addition it is possible to control and evaluate the students in a more efficient and complete way than the evaluation made in past time; this can improve the processes of training and reach more satisfactory results by the students.

## Figures and Tables

**Table 1:** Relation about key aspects in the training process and checkpoints that we need to know using the cloud-based analysis system.

Items to audit	Checkpoints monitored by cloud system
Laboratory access	Entry across the SAS
Installations	Entrance to all rooms
Equipment	Revision of the equipment documentation (calibration, qualification, cleaning and maintenance, etc.)
Emergency systems	Check of shower and eyebaths
	Check of emergency door, extinguisher, and the medicine chest
Documentation	SOPs (standard operating procedures)
	File cabinet of Research and Development documentation (locked)

**Table 2:** Evaluation of the training performed in Usalpharma Lab.

Evaluated aspects		Mark
From data saved in the cloud architecture	Activity control (i) Activity dates (ii) Number and duration of the lab access	Requirement to pass (i) Work in the activity period (dates fixed previously by teachers) (ii) Minimum total duration of 1 hour
	Audit methodology compliance (i) Executed actions show in a list and as a percentage (ii) Nonexecuted actions	25% of total mark (minimum required of 12.5%)

From the audit report	Ability to (i) identify regulation deviations (ii) refer the deviations to the specific regulations aspect (iii) classify the deviations depending on their criticality	60% of total mark
	Verdict about the virtual laboratory quality system	15% of total mark
